# Genomic features of lichen‐associated black fungi

**DOI:** 10.1002/iub.2934

**Published:** 2024-12-22

**Authors:** Victoria Keller, Anjuli Calchera, Jürgen Otte, Imke Schmitt

**Affiliations:** ^1^ Senckenberg Biodiversity and Climate Research Centre (S‐BiKF), Senckenberg Gesellschaft für Naturforschung Frankfurt am Main Germany; ^2^ Institute of Ecology, Evolution and Diversity Goethe University Frankfurt am Main Frankfurt am Main Germany; ^3^ LOEWE Centre for Translational Biodiversity Genomics (LOEWE‐TBG) Frankfurt am Main Germany

**Keywords:** biosynthetic gene clusters, diversity, funannotate, melanin, PacBio, phylogenetics, symbiosis

## Abstract

Lichens are mutualistic associations consisting of a primary fungal host, and one to few primary phototrophic symbiont(s), usually a green alga and/or a cyanobacterium. They form complex thallus structures, which provide unique and stable habitats for many other microorganisms. Frequently isolated from lichens are the so‐called black fungi, or black yeasts, which are mainly characterized by melanized cell walls and extremophilic lifestyles. It is presently unclear in which ways these fungi interact with other members of the lichen symbiosis. Genomic resources of lichen‐associated black fungi are needed to better understand the physiological potential of these fungi and shed light on the complexity of the lichen consortium. Here, we present high‐quality genomes of 14 black fungal lineages, isolated from lichens of the rock‐dwelling genus *Umbilicaria.* Nine of the lineages belong to the Eurotiomycetes (Chaetothyriales), four to the Dothideomycetes, and one to the Arthoniomycetes, representing the first genome of a black fungus in this class. The PacBio‐based assemblies are highly contiguous (5–42 contigs per genome, mean coverage of 79–502, N50 of 1.0–7.3 mega‐base‐pair (Mb), Benchmarking Universal Single‐Copy Orthologs (BUSCO) completeness generally ≥95.4%). Most contigs are flanked by a telomere sequence, suggesting we achieved near chromosome‐level assemblies. Genome sizes range between 26 and 44 Mb. Transcriptome‐based annotations yielded ~11,000–18,000 genes per genome. We analyzed genome content with respect to repetitive elements, biosynthetic genes, and effector genes. Each genome contained a polyketide synthase gene related to the dihydroxynaphthalene‐melanin pathway. This research provides insights into genome content and metabolic potential of these relatively unknown, but frequently encountered lichen associates.

**Abbreviations**
BF: black fungus
BLAST: Basic Local Alignment Search Tool
BS: bootstrap
BUSCO: Benchmarking Universal Single‐Copy Orthologs
DHN: dihydroxynaphthalene
FAS: fatty acid synthase
HiFi: high‐fidelity
ITS: internal transcribed spacer
KS: keto synthase
Mb: mega‐base‐pair
MIBiG: Minimum Information about a Biosynthetic Gene cluster
ML: maximum likelihood
nr: non‐redundant
NR‐PKS: non‐reducing polyketide synthase
NRPS: non‐ribosomal peptide synthetase
nuLSU: large subunit (nuclear)
PacBio: Pacific Biosciences
PCR: polymerase chain reaction
PKS: polyketide synthase
RiPP: ribosomally synthesized and post‐translationally modified peptide
SMRT: single‐molecule real‐time

## 1 INTRODUCTION

Lichens are obligate symbiotic associations of a primary fungal host (mycobiont) and one or more green algal and/or cyanobacterial photosynthetic symbionts (photobiont).^1^ However, this description of the lichen consortium is too simplistic, and today, lichens are considered as microecosystems^2^ hosting diverse microorganisms, including bacteria,^3^, ^4^, ^5^, ^6^ further algae,^7^, ^8^ and fungi.^9^, ^10^ In fact, lichen‐infecting fungi were described even before lichens were recognized as a mutualistic association.^11^ These so‐called lichenicolous fungi have been known for a long time, as they are symptomatic, forming fruiting bodies or other visible structures on the lichen thalli.^12^ In addition, there are asymptomatically occurring fungi, which are described as growing endolichenically, including the so‐called black fungi.^9^, ^13^ In the past two decades, lichen‐associated black fungi have gained increasing scientific attention.^14^, ^15^, ^16^, ^17^

Many black fungi, such as *Hortaea werneckii*,^18^ *Coniosporium apollinis*,^19^, ^20^ or *Trimmatostroma salinum*,^18^, ^21^ are considered extremotolerant and/or extremophilic, as they can thrive under environmental conditions, which preclude the survival of most other organisms.^22^ They can be isolated from a variety of substrates^23^, ^24^, ^25^, ^26^, ^27^, ^28^, ^29^, ^30^, ^31^, ^32^, ^33^, ^34^ and are characterized by their morphological and physiological characteristics rather than their monophyletic origin.^26^ The majority of black fungi belong to different classes of Ascomycota, including Dothideomycetes, Eurotiomycetes,^14^, ^15^ Sordariomycetes,^17^, ^35^ and Arthoniomycetes.^36^ Their eponymous feature is the dark‐brown to black pigmentation, attributed to the constitute biosynthesis of melanins.^37^

Melanins have diverse ecological functions, including environmental stress tolerance, pathogenicity and virulence, and protection against microbial attacks.^37^, ^38^, ^39^ Abiotic environmental tolerances conferred by melanin biosynthesis include protection against radiation and extreme temperatures,^40^ desiccation,^41^ and acidic environments.^42^ In addition to providing protection from abiotic factors, melanins are also important in mediating biotic interactions, such as invasion of hosts,^43^ and protection against lysis and phagocytosis.^44^ However, many of the proposed functions of melanins remain uncertain, as indicated, for example, by albino strains, which—in some cases—show no less resistance to stress factors than melanotic strains.^45^, ^46^, ^47^ Fungi synthesize structurally diverse melanin polymers via different biosynthetic pathways, including the 1,8‐dihydroxynaphthalene (DHN) pathway, the L‐3,4‐dihydroxyphenylalanine (L‐dopa) pathway,^48^ the L‐tyrosine degradation pathway,^49^ a pathway involving a non‐ribosomal peptide synthetase‐like enzyme that produces aspulvinone E,^42^ and a polyketide pathway involving coumarin and pyran‐2‐one intermediates.^50^ However, genes putatively associated with melanin biosynthesis have not yet been reported from black fungi isolated from lichens.

Black fungi and lichenized fungi share the same habitats, for example, rock surfaces, where they are both found in great diversity.^20^ Thus, it may not be surprising that black fungi are frequently isolated from rock‐dwelling lichens. However, it is unclear to what extent they interact with other members of the lichen symbiosis. Some species even appear to be lichen‐specific; that is, they have frequently been isolated from lichens, but so far never from other substrates.^17^ It is therefore possible that some species of black fungi might directly benefit from growing inside lichens,^20^, ^51^, ^52^ for example, by consuming photo‐assimilates provided by phototrophic lichen symbionts. Contrarily, it is also possible that the main lichen partners—the mycobiont and photobiont—profit from advantages conferred by the melanin biosynthesis of black fungi. Indeed, lichen‐associated black fungi are capable of releasing dark pigments into their immediate environment, as evidenced by cultures exuding pigments into the substrate.^53^ In general, it is difficult to visualize and study these cryptically occurring fungi inside their lichen hosts, and microscopy methods and various experimental approaches, for example, fluorescence in situ hybridization (FISH)^54^, ^55^, ^56^ face technical limitations. On the other hand, co‐culturing of axenically growing black fungi with lichen‐forming algae has previously shown visible attachments that later on resulted in lichen‐like structures.^57^ Whether such connections develop inside the lichen has not been clarified yet.

Genome analyses hold great promise to shed light on the evolution of fungal lifestyles^58^, ^59^ and other fungal traits, such as secondary metabolism.^60^ Genome comparisons will help us to uncover common genomic features and eventually link distinct genomic features to complex traits,^61^ including the polyextremophile and ecological specialization of black fungi.^62^ However, genomic diversity is unevenly sampled across the fungal phylogeny, and many lineages, including specialists to particular ecological niches, are insufficiently represented in genomic databases.^63^

Here, we set out to establish the first genomic resource of lichen‐associated black fungi, consisting of highly contiguous and complete genomes. To this end, we sequenced 14 phylogenetically diverse black fungal lineages isolated from rock‐dwelling lichens of the genus *Umbilicaria*. Specifically, we asked the following questions: (1) What is the phylogenetic placement of the black fungal isolates of the present study, based on multilocus phylogenies of the internal transcribed spacer (ITS) and nuclear large subunit (nuLSU)? (2) What are the basic genomic features of black fungi isolated from lichens, for example, genome size, number of chromosomes, number of genes, etc.? (3) What are the diversity of biosynthetic genes and the amount of effector genes? (4) Are there homologous polyketide synthase genes shared by lichen‐associated black fungi that are putatively linked to melanin biosynthesis?

## 2 MATERIALS AND METHODS

### 2.1 Isolation and culturing

We isolated black fungi from the rock‐dwelling, lichen‐forming species *Umbilicaria phaea*, *U. pustulata*, *U. crustulosa*, and *U. nylanderiana*. All lichen specimens had been air‐dried after collecting and stored at −20°C. Collection information about the lichen samples is provided in Table S1. To isolate lichen‐associated black fungi, we followed protocols originally developed for isolating lichen mycobionts and photobionts,^64^, ^65^ with a few modifications. Briefly, we cut small (~1–2 mm^2^) thallus pieces and washed those for 3 h under a constant flow of distilled water instead of Tween. To remove any access water after washing, samples were air‐dried on Whatman 3MM (Cytiva, Marlborough, MA, USA) blotting paper for a few minutes. Thallus fragments were carefully placed atop a small dab of Vaseline within the lid of a Petri dish, then covered with the bottom of the dish containing the medium. We used Malt‐Yeast agar^66^ for isolation and cultivation, with 1.5% agar. Thallus pieces were removed after 3–5 days of exposing under the sterile bench and Petri dishes were sealed. Cultures were incubated at 20–22°C in the dark and regularly checked for contaminations and growth. Black fungal cultures were visibly evident after approximately 1 month and were immediately transferred onto new plates. We generated an ITS ribosomal DNA (rDNA) sequence to check fungal identity and obtain a rough phylogenetic placement of the strain. Each time we found a strain that had a different ITS sequence from the strains we had isolated before, we selected it for genome sequencing. We ended up with 14 phylogenetically distinct lineages, which we subjected to de novo genome sequencing.

### 2.2 DNA extraction, ITS, and nuLSU sequencing

DNA was extracted using the Quick‐DNA Fungal/Bacterial Miniprep Kit (Zymo Research Europe GmbH, Freiburg, Germany) or cetyltrimethylammonium bromide protocol.^67^ The ITS and nuLSU were amplified using the primers ITS1F/ITS4^68^, ^69^ and LR0R/LR5.^70^, ^71^ Each 12‐μL polymerase chain reaction (PCR) contained 10 ng of genomic DNA (gDNA) template, 6‐μL hot start polymerase (MyTaq HS Mix, 2x, Meridian Bioscience Inc., Cincinnati, OH, USA), 0.4 μL for each of the 10‐μM primers, and 4.2 μL of PCR grade H_2_O. PCR amplifications were performed under the following conditions: One initial denaturation step of 1 min at 95°C, followed by 35 cycles of denaturation at 95°C for 15 s, annealing at 52°C for 15 s, and extension at 72°C for 10 s. PCR products were checked for their quality and size by 1% agarose gel electrophoresis and sequenced using the BigDye Terminator v3.1 Cycle Sequencing Kit (Applied Biosystems, Foster City, CA, USA) under the following conditions: initial denaturation for 1 min at 95°C, followed by 30 cycles of 96°C for 10 s, 50°C for 10 s, and 60°C for 2 min. The products were purified using the BigDye XTerminator Purification Kit (Life Technologies, Foster City, CA, USA) and detected on an ABI PRISM 3730 DNA Analyzer (Applied Biosystems, Foster City, CA, USA). Preliminary identifications of the isolates were done using the Basic Local Alignment Search Tool (BLAST).^72^ For simplicity reasons, samples were abbreviated with “BF” (black fungus [BF]) and numbered.

### 2.3 High molecular weight DNA extraction and PacBio sequencing

Genomic DNA extraction and library preparation were done as described in Ahmad et al.^73^ Briefly, gDNA of the black fungi was extracted using the Quick‐DNA Fungal/Bacterial Miniprep Kit (Zymo Research, Europe GmbH, Freiburg, Germany). Due to the presence of inhibiting substances, for example, pigments, further purifications were carried out utilizing the Genomic DNA Clean & Concentrator‐10 kit (Zymo Research, Europe GmbH, Freiburg, Germany) and/or the DNeasy PowerClean Cleanup Kit (Qiagen, Venlo, the Netherlands). Quality assessments of gDNAs were performed with the Implen GmbH NanoPhotometer Pearl (Munich, Germany), TapeStation 2200 (Agilent Technologies Inc., Santa Clara, CA, USA), and Qubit 2.0 Fluorometer (Thermo Fisher Scientific, Waltham, MA, USA). Only for samples passing quality control (260/280 absorbance ratio of 1.75–1.85, 260/230 absorbance ratio of 2.0–2.2), SMRTbell (single‐molecule real‐time) libraries were prepared, following the Low DNA Input Protocol of the SMRTbell Express Template Prep Kit 2.0 (Pacific Biosciences [PacBio] of California, Inc., Menlo Park, CA, USA), with a T‐overhang SMRTbell adapter ligation time of 1 h. The purification with AMPure PB beads was carried out twice. Using the TapeStation 2200 (Agilent Technologies Inc., Santa Clara, CA, USA) and Qubit 2.0 Fluorometer (Thermo Fisher Scientific, Waltham, MA, USA) with the Qubit dsDNA‐HS Assay Kit (Thermo Fisher Scientific, Waltham, MA, USA), the final concentration was assessed.

Libraries were sent to the Genome Technology Center (RGTC) of the Radboud University Medical Center (Nijmegen, the Netherlands) for SMRT sequencing as described in Merges et al.^74^ with the difference that two barcoded genomes (Barcoded overhang adapter kit 8A, PacBio of California, Inc., Menlo Park, CA, USA) were multiplexed and sequenced in “circular consensus sequencing” mode on one SMRT cell.

### 2.4 RNA sequencing

We used the TRI Reagent (Zymo Research, Europe GmbH, Freiburg, Germany) for RNA extraction following the manufacturer's protocol (Zymo Research, Europe GmbH, Freiburg, Germany). About 60–80 mg of BF mycelium was ground to a fine powder in a mortar, using liquid nitrogen. The powder was then transferred into a 2‐mL Eppendorf tube (Eppendorf SE, Hamburg, Germany). 1.2 mL of the TRI Reagent (Zymo Research, Europe GmbH, Freiburg, Germany) was added and mixed well by vortexing. The lysate was centrifuged in an Eppendorf Centrifuge 5424R (Eppendorf SE, Hamburg, Germany) with a FA‐45‐24‐11 Eppendorf Rotor (Eppendorf SE, Hamburg, Germany) for 5 min at 12,000 × *g*, 4°C. The clear supernatant was transferred to a new tube. After extraction and precipitation, the RNA was dissolved in 55 μL RNase‐free water. Quality and quantity of the RNA extracts were assessed spectrophotometrically using a NanoPhotometer P300 (Implen GmbH, Munich, Germany). The purification was performed using the RNA Clean & Concentrator‐5 kit (Zymo Research, Europe GmbH, Freiburg, Germany). We purified the RNA samples as many times as needed until reaching a 260/280 absorbance ratio of 1.9–2.1 and a 260/230 absorbance ratio of 1.8–2.2 and an RNA integrity number >8.0. RNA was sent to Novogene (Cambridge, England) for a non‐stranded library preparation and paired‐end sequencing (150 base pair) on the Illumina NovaSeq platform.

### 2.5 Phylogenetic analyses for species identification

Due to insufficient genomic reference taxa in public databases, we decided to perform the phylogenetic placement of the lichen‐associated black fungal strains based on ITS and nuLSU. Preliminary phylogenies showed that our samples grouped with three classes of the Ascomycota: Eurotiomycetes, Dothideomycetes, and Arthoniomycetes, leading us to create separate alignments for each class. The alignment of Eurotiomycetes is based on recently published phylogenies, Cometto et al.,^17^ Quan et al.,^75^ and Carr et al.,^76^ mostly including vouchers of which the ITS and nuLSU locus are available in GenBank.^77^ All sequences in this alignment belong to the Chaetothyriales. We selected outgroup taxa according to Quan et al.^75^ The alignment of Dothideomycetes is based on Cometto et al.^17^ and Egidi et al.^78^ with outgroup sequences as in Cometto et al.^17^ The alignment of the Arthoniomycetes is based on Muggia et al.^36^ This alignment is much smaller than the other two, as not many species of Arthoniomycetes are available in GenBank,^77^ for which both ITS and nuLSU are available. We supplemented all three alignments with further sequences, which showed high similarity to strains analyzed here, based on Nucleotide BLAST (blastn)^72^ searches in the standard core nucleotide database (core_nt).

Sequences were aligned using MAFFT v.7^79^ with the G‐INS‐i iterative refinement method. Using Molecular Evolutionary Genetics Analysis v.7,^80^ ends were trimmed and ambiguous sequences were removed from the alignments. Concatenation of the two loci was done using Geneious Prime v23.0.2 (https://www.geneious.com). A maximum likelihood (ML) phylogenetic analysis of each phylogeny was performed using IQTree 2.2.5^81^ with 1000 replicates for ultrafast bootstrap (BS) (−B)^82^ to assess branch support. Respective outgroups (−o) were set. To detect the most appropriate model, ModelFinder (−m MFP)^83^ was set, calculating and choosing TIM2e+I+R6 as best‐fit model for the Chaetothyriales alignment, TIM2e+I+R4 for the Dothideomycetes, and TIM2+F+I+G4 for the Arthoniomycetes alignment. Phylogenetic trees were visualized using ITOL v6.8.2^84^ and edited in Inkscape v1.3.2 (091e20e, 2023‐11‐25, custom) (The Inkscape Team 2023, https://inkscape.org). Only BS support values ≥70% are shown. In each phylogenetic tree, we collapsed all homologous clades that did not contain any of the black fungal strains analyzed here. One exception was made in the Dothideomycetes alignment for the clade of the Teratosphaeriaceae. The full phylogenetic trees and sequence accessions are available as supplemental material (Figures [Link], [Link], Table S2).

### 2.6 Genome assembly

The genomes were assembled using PacBio high‐fidelity (HiFi) long reads. Raw subreads were filtered and HiFi reads were created using pbccs v6.4.0 with default settings, which is implemented in the pbbioconda package (PacBio 2022, https://github.com/PacificBiosciences/pbbioconda). Another implementation of this package, Lima v2.7.1 (PacBio 2022, https://github.com/PacificBiosciences/pbbioconda), was used for demultiplexing of the samples and removing the barcodes. A combination of parameters was set according to the recommendations for symmetric data (PacBio 2022, https://github.com/PacificBiosciences/pbbioconda). Using the BamTools toolkit v2.5.2,^85^ the quality of the reads was checked, and the data were converted for further downstream analyses. De novo assemblies were generated utilizing the Flye assembler v2.9.1^86^ with parameters set for PacBio reads. For taxonomic binning, we followed the instructions of the DIAMOND v2.0.15.153^87^ + MEGAN v.6.24.16 pipeline after Huson et al.^88^ aligning DNA reads against the NCBI‐nr (non‐redundant) v5 protein reference database^89^ (downloaded February 2023). For functional analyses and mapping of the NCBI‐nr accessions to taxonomic classes, the megan‐map database version Feb2022^88^, ^89^ (https://software-ab.cs.uni-tuebingen.de/download/megan6/welcome.html) was downloaded and used. If necessary, contaminating or non‐assigned sequences were removed from the assemblies by extracting correctly assigned reads from the respective nodes in MEGAN v6.24.16.^88^

A quality check of the assemblies was done by checking the genome completeness according to Benchmarking Universal Single‐Copy Orthologs (BUSCO) v5.4.5^90^ and applying ITSx v1.1.3^91^ to screen for different ITS sequences in the assemblies. BUSCO^90^ lineage datasets were chosen according to the most complete, yet phylogenetically closest related group (Table S3). Lastly, we used Tapestry v1.0.1^92^ to detect and visualize conserved telomeric repeats (TTAGGG/CCCTAA)^93^, ^94^ on contigs (Figure S4).

### 2.7 Genome annotation

All genomes were annotated using the sequenced RNA transcripts in the funannotate pipeline v1.8.15.^95^ Duplicated contigs of the assemblies were removed, contigs were sorted by size, and repeat masked using the funannotate commands “clean,” “sort,” and “mask” with default parameters.^95^ After running the assemblies through the training and prediction pipeline, incorporating RNAseq data, and the most complete BUSCO gene sets,^90^ gene predictions were updated and gene models fixed. Due to the expected high gene density of the fungal genomes, the ‐jaccard_clip option was set when running funannotate “train” and “update”.^95^ We also predicted secreted proteins, effector genes, and secondary metabolite gene clusters using SignalP v6.0,^96^ EffectorP v3.0.0,^97^ and antiSMASH v7.1.0,^98^ respectively. EffectorP^97^ was run in fungal mode. Parameters set for antiSMASH v7.1.0 were the relaxed Hidden Markov Model‐based cluster detection, and all parameters for additional analyses.^98^ Furthermore, InterProScan v1.8.15^99^, ^100^ was run to classify protein families. Funannotate “annotate” was finally utilized for the functional annotation of the genes^95^ including parameters for the most complete BUSCO gene sets,^90^ and all data generated during antiSMASH,^98^ SignalP,^96^ and InterProScan^99^, ^100^ analyses. Repetitive elements of the 14 black fungal genomes were identified using RepeatModeler v2.0.2 (Smit, AFA, Hubley, R, see http://www.repeatmasker.org), followed by RepeatMasker v4.1.2 (Smit, AFA, Hubley, R & Green, P., see http://www.repeatmasker.org).

### 2.8 Phylogenetic analysis of Ascomycota polyketide synthases

We performed a phylogenetic analysis of the conserved keto synthase (KS) domain of predicted and annotated type I polyketide synthase (PKS) gene(s) for all black fungal isolates using data of the Minimum Information about a Biosynthetic Gene cluster (MIBiG)^101^, ^102^ database. We retrieved all available KS sequences of Ascomycota from the database to align the detected KS domains of our samples focusing on the non‐reducing PKSs (NR‐PKSs) (Figure 4). We used six fatty acid synthase (FAS) outgroup sequences available in GenBank^77^ according to Calchera et al.^103^ (Table S4) that were summarized as “Outgroup Sequences” in Figure 4. Amino acid sequences were aligned, ML phylogenetic analyses performed, and trees were visualized and edited as described in section 2.5. The best‐fit model detected for the NR‐PKS phylogeny was Q.pfam+R5. Clades were numbered and named according to Kim et al.,^104^ Gerasimova et al.,^105^ and Ebert et al.^106^ A phylogeny including all PKSs (best‐fit model Q.pfam+R5) is available as Figure S5 with detailed sequence information in Table S4.

## 3 RESULTS AND DISCUSSION

### 3.1 Phylogenetic placement of black fungal lineages

Species identification of black fungi can be challenging without any DNA sequencing, as their axenic cultures possess only few—sometimes too few—distinct taxonomic features.^20^ Here, we analyzed combined ITS and nuLSU phylogenies to obtain a phylogenetic placement of the 14 black fungal lineages.

**FIGURE 1:**
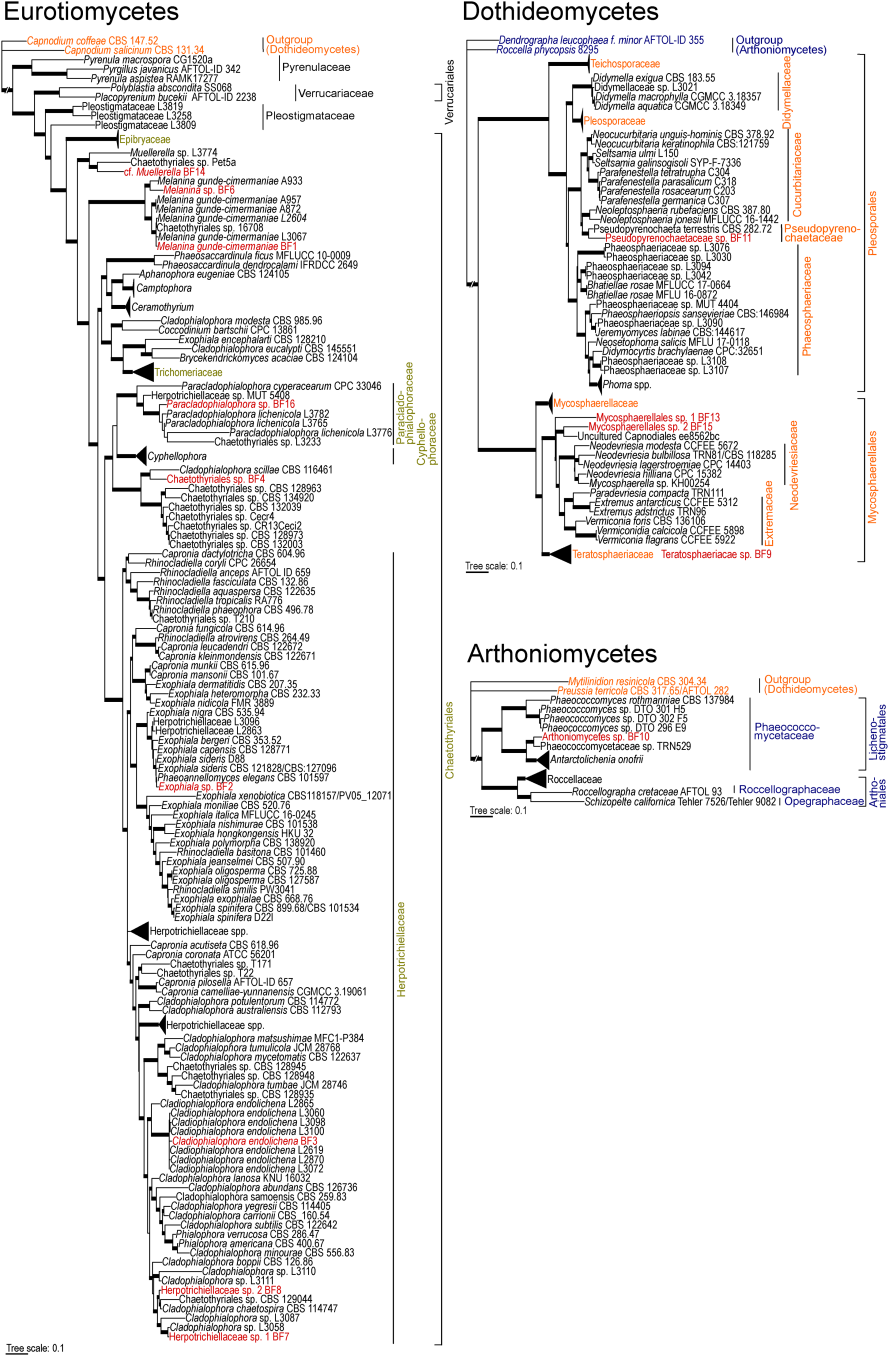
Phylogenetic placement of 14 black fungal lineages isolated from lichens. The new lineages are highlighted in red and group in the Eurotiomycetes, Dothideomycetes, and Arthoniomycetes. Presented trees are maximum likelihood phylogenies based on concatenated ITS and nuLSU sequences. Branches in bold indicate ML bootstrap support ≥70%. All data retrieved from GenBank^77^ are provided in Table S2. Full phylogenetic trees, without collapsed clades and detailed BS, are available as Figures [Link], [Link].

The ML phylogenetic trees of Eurotiomycetes, Dothideomycetes, and Arthoniomycetes generated in this study are highly concordant in their topology with previous studies.^17^, ^36^, ^75^, ^78^, ^107^, ^108^ Most clades are supported with a BS support value ≥70% (Figure 1, Figures [Link], [Link]).

The Eurotiomycetes phylogeny comprises 242 sequences, including the nine newly sequenced black fungal isolates (Figure 1). We assigned taxon names to the newly isolated strains based on their phylogenetic placements. If a strain grouped with high BS support in a clade consisting of known species, we assigned the respective name. If a strain was not assignable to a species, we assigned it to the next higher, confidently supported level, of either genus, family, or order. All of the newly isolated Eurotiomycetes belong to the Chaetothyriales. Two strains in this order, BF1 and BF3, could phylogenetically be assigned at the level of species. BF1 can be assigned to the species *Melanina gunde‐cimermaniae*. The recently described genus *Melanina* contains only this species and forms a distinct lineage within the Chaetothyriales.^16^ Isolates of this taxon were obtained from rock‐dwelling lichens,^16^, ^17^, ^35^, ^53^ including another species of *Umbilicaria*,^16^ as well as from rocks.^109^ BF3, *Cladophialophora endolichena*, was recently described as a strictly lichen‐associated BF, which has been isolated from a number of lichen genera (*Lecanora*, *Protoparmeliopsis*, *Rhizoplaca*, *Tephromela*), originating from different continents (Australia, Europe, North and South America) and environments.^17^ Our finding, which is based on an isolate from the genus *Umbilicaria*, supports the observation that this fungus is frequently associated with lichens growing on acidic rocks, and possibly—since it has never been isolated from substrates other than lichens—has its realized ecological niche within lichen thalli.^17^

Four strains could be assigned at the level of genus, including BF6 (*Melanina*), BF2 (*Exophiala*), BF16 (*Paracladophialophora*), and tentatively BF14 (cf. *Muellerella*). The present phylogeny shows two strongly supported clades within *Melanina*, indicating that there is either intraspecific variation within *M. gunde‐cimermaniae* or that the genus contains more than one species. We decided to call the slightly distinct genome‐sequenced lineage of BF6 *Melanina* sp. Species of the genus *Muellerella* are commonly recognized as symptomatically growing lichenicolous fungi, forming visible perithecia,^110^, ^111^ but have also been observed growing cryptically within lichens.^17^ The present isolate (BF14) is sister to a member of Chaetothyriales,^112^ which was later assigned to the genus *Muellerella*.^17^, ^111^ *Muellerella* is polyphyletic within the Chaetothyriomycetidae.^111^ BF14 clusters in the clade “*Muellerella* + *Lichenodiplis*” according to Muggia et al.^17^, ^110^, ^111^ As there are not many specimens sequenced at both, the ITS and nuLSU locus, the present clade comprises only three sequences.^17^, ^112^ We therefore decided to tentatively assign BF14 to this group as “cf. *Muellerella*”. BF2 groups with several other taxa of *Exophiala* and was assigned to this taxon, as *Exophiala* sp. Some members of *Exophiala* belong to biological soil crust consortia and may be capable of utilizing carbon and nitrogen sources potentially derived from symbiotic microbes.^76^ Other species of this genus were isolated from diverse substrates, for example, leaves,^113^ different anthropogenic environments,^27^ and lichens.^17^ Possibly, members of this taxon rely on photo‐assimilates provided by symbiotic microbes, plants, or lichen‐symbiotic green algae for their own nutrition. BF2 clusters within a clade that includes two isolates, “Herpotrichiellaceae” (L3096) and “Herpotrichiellaceae” (L2863), that were obtained from the lichenized fungi *Rhizoplaca melanophthalma* and *Tephromela atra*.^17^ Lineage BF16 grouped with several strains belonging to the recently described species *Paracladophialophora lichenicola*.^17^ This species was recurrently isolated from different species of lichenized fungi, including another species of the genus *Umbilicaria*.^17^ Since *P. lichenicola* has never been found on other substrates, it is presumed to be lichen‐specific.^17^ However, as BF16 clusters basally to the *P. lichenicola* strains, we decided to assign this specimen preliminarily only at genus level.

Three samples could phylogenetically only be assigned at the family or order level, including BF4 (Chaetothyriales), BF7, and BF8 (both Herpotrichiellaceae). BF4 groups with *Cladophialophora scillae* ^114^ and forms the sister group to several strains designated as Chaetothyriales sp. that are, according to Vasse et al.^112^ assigned to Cyphellophoraceae. Interestingly, all close relatives are isolates from ant occupied plant domatia,^112^, ^115^ except *C. scillae* that was phytopathogenically growing on *Scilla peruviana*.^114^ The entire clade forms the sister group of Cyphellophoraceae and Paracladophialophoraceae. As the latter was not described until 2018, the family concept of the present clade potentially has to be revised.^116^ BF7 and BF8 are grouping within sister clades of each other. Only the clade of BF7 summarizes two isolates from lichens, both are assigned as *Cladophialophora* sp. (L3087, L3058).^17^

Throughout the Chaetothyriales phylogeny, we observed that the newly sequenced black fungi are often closely related to other fungi isolated from lichens (BF1, BF2, BF3, BF6, BF7, BF16) or to fungi isolated from domatia (BF4, BF8, BF14). Domatia are diverse hollow structures, produced by ant‐plants, to provide nesting sites to their ant partners.^117^ These structures are typically overgrown by a thin layer of fungal mycelium, mainly constituted of Chaetothyriales strains,^118^ and are recognized as a mutualistic association between plant, ant, and fungus.^119^, ^120^ The phylogenetic relationships observed here might indicate that certain strains of black fungi profit from living in complex symbiotic communities, or might even depend on conditions provided by such consortia.

The alignment of Dothideomycetes, comprising 111 taxa—including four from the current study—represents only Pleosporales and Mycosphaerellales (Figure 1). The black fungal isolates clustering into the Dothideomycetes are widely distributed across the phylogeny. This heterogenous and rather scattered phylogenetic distribution of black fungi within the Dothideomycetes has previously been observed several times.^14^, ^17^ We could assign the new sequences only at the level of family or order. Within the Pleosporales, BF11 aligns with a sequence of *Pseudopyrenochaeta terrestris*, isolated from soil.^121^ The remaining three black fungal isolates are clustering within the Mycosphaerellales: BF13, Mycosphaerellales sp. 1, groups basally to the Extremaceae and Neodevriesiaceae. BF15, Mycosphaerellales sp. 2, groups with an “Uncultured Capnodiales” (Furneaux, R. B., GenBank^77^ accession: HG996345) sequence, isolated from soil, as sister group to the Neodevriesiaceae. The fourth isolate, BF9, Teratosphaeriaceae sp., could be assigned to the Teratosphaeriaceae, which builds a greatly supported clade (BS 100%) in the phylogeny. The sequence of BF9 groups with another sequence assigned as Teratosphaeriaceae sp. (L3239),^17^ which was isolated from a lichen^17^ (Figure S2). Interestingly, compared to the black fungal isolates belonging to the Eurotiomycetes, we could not find any close lichen‐associated relatives to our isolates within the Dothideomycetes phylogeny, except for BF9.

For the phylogenetic placement of the only Arthoniomycete genome, we aligned 95 sequences, 13 belonging to *Antarctolichenia onofrii* ^36^ (Figure 1). The phylogeny outlines the phylogenetic relationship of the two sister clades Arthoniales and Lichenostigmatales, representing five different families of the Arthoniomycetes. While most Arthoniales are lichenized taxa, Lichenostigmatales mainly include rock‐inhabiting fungi, black yeasts, and most of all, lichenicolous fungi.^122^ Our isolate groups with a sequence described as Phaeococcomycetaceae sp. (BS 99%) that was isolated from a rock surface,^123^ within the greatly supported family clade of Phaeococcomycetaceae (BS 100%) (Lichenostigmatales) (Figure S3). In general, sequences of the Arthoniomycetes are highly underrepresented in public sequence databases such as GenBank,^77^ especially regarding the ITS locus.^36^ We therefore tentatively called the isolate BF 10 Arthoniomycetes sp.

### 3.2 Genome assembly and annotation

**FIGURE 2:**
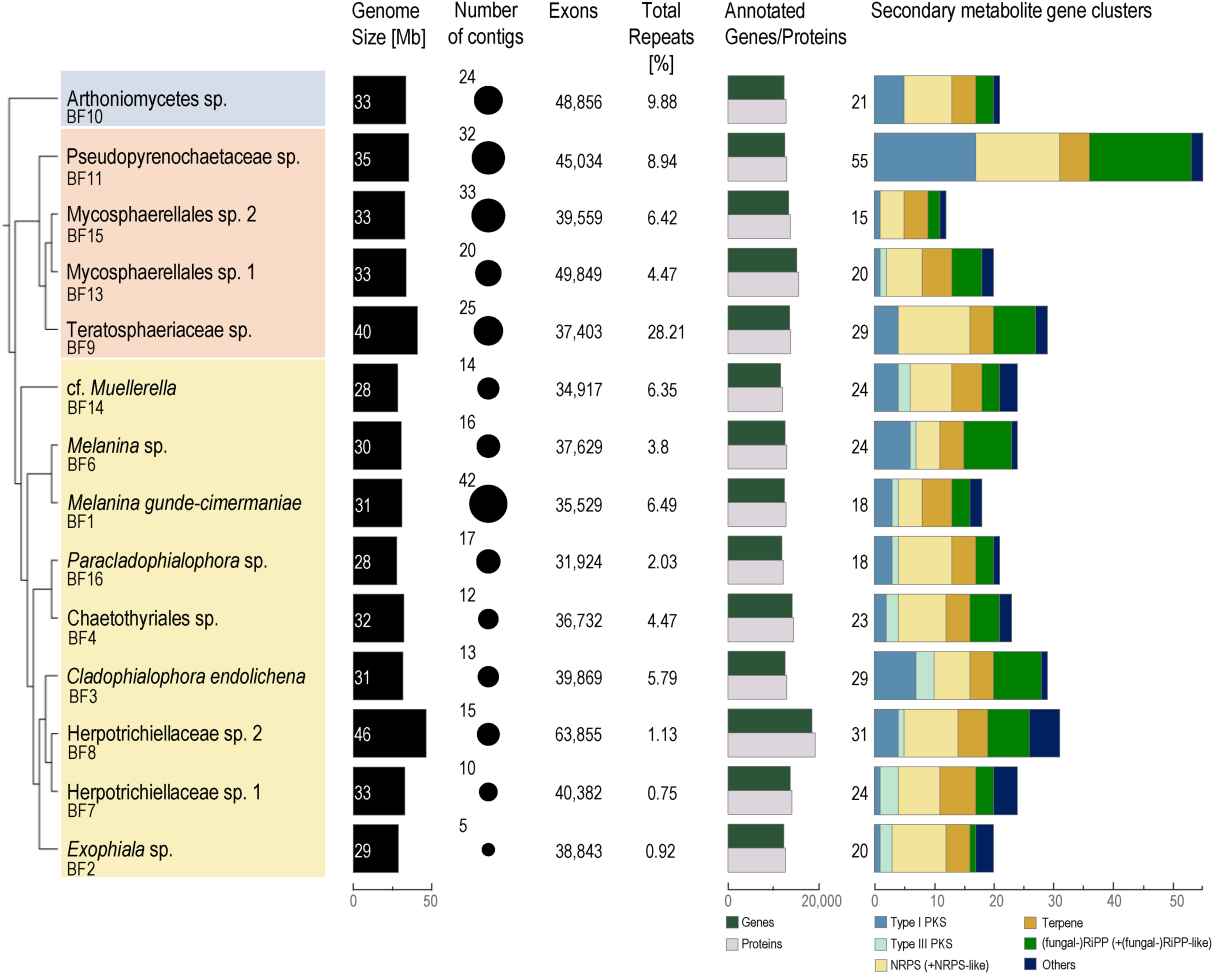
Genome assembly and annotation statistics of 14 de novo genomes of black fungi isolated from lichens. Indicated are genome size, number of contigs, number of exons, percentage of repeats, number of annotated genes and proteins, and composition of secondary metabolite gene clusters. Phylogenetic relationships of the analyzed taxa are indicated (yellow—Eurotiomycetes, orange—Dothideomycetes, blue—Arthoniomycetes). Secondary metabolite categories comprise the following: type I and III PKS, NRPS and NRPS‐like, terpenes, (fungal‐)ribosomally synthesized and post‐translationally modified peptides (RiPPs) and (fungal‐)RiPP‐like, and others. Others include phosphate‐like, betalacetones, indoles, non‐alpha poly‐amino acids, and non‐ribosomal peptide‐metallophores (NRP‐metallophore). Secondary metabolite gene clusters were identified using antiSMASH.^98^ A detailed overview on genome assembly and annotation statistics is provided in Table S3.

Genome sizes ranged between 27.5 and 45.9 mega‐base‐pair (Mb), with 5–42 contigs per genome, and an average contig N50 of 3.1 Mb, ranging between 1 and 7.3 Mb (Figure 2, Table S3). Contigs had a mean coverage of 79–502, and genomes had a GC content between ~48%–52% (Table S3). BUSCO completeness was ≥95.4% with a duplication rate ≤0.8% in all samples, except *Paracladophialophora* sp. (BF16), where completeness was only 92.9% (Table S3). This however may be due to the fact that the entire clade of BF16 encompasses relatively unknown species, and in this regard, the BUSCO reference database may be incomplete.

The detection of telomeric repeats on the ends of each contig provides an overview on the completeness of genomes (Table 1). In our case, most contigs are flanked by a telomere sequence on both ends, or at least one end (Figure S4). In some of the assembled genomes almost all contigs are framed with telomeric repeats, allowing an estimate of the number of chromosomes in these species, for example, *Exophiala* sp. (BF2)—5 chromosomes, *Melanina* sp. (BF6)—14 chromosomes, and Herpotrichiellaceae sp. 1 (BF7)—8 chromosomes. The number of chromosomes most likely varies between 4 and 30 for all 14 black fungal lineages (Table 1), although, for example, karyotyping is needed to confirm the exact number of chromosomes for each genome.

**TABLE 1:** Genome contiguity of 14 de novo genomes of black fungi isolated from lichens.

ID	Name	Number of contigs	Number of telomeres	No. of contigs assembled T–T
BF10	Arthoniomycetes sp.	24	39	16
BF11	Pseudopyrenochaetaceae sp.	32	47	19
BF15	Mycosphaerellales sp. 2	33	60	27
BF13	Mycosphaerellales sp. 1	20	26	9
BF9	Teratosphaeriaceae sp.	25	41	18
BF14	cf. *Muellerella*	14	22	9
BF6	*Melanina* sp.	16	28	13
BF1	*Melanina gunde‐cimermaniae*	42	37	13
BF16	*Paracladophialophora* sp.	17	18	5
BF4	Chaetothyriales sp.	12	21	9
BF3	*Cladophialophora endolichena*	13	10	2
BF8	Herpotrichiellaceae sp. 2	15	7	0
BF7	Herpotrichiellaceae sp. 1	10	17	7
BF2	*Exophiala* sp.	5	9	4

*Note*: Number of contigs, telomeres, and contigs assembled telomere‐to‐telomere for each genome assembly are given. Telomeres were detected using Tapestry.^92^

The repeat content was variable among the genomes, ranging from 0.75% in Herpotrichiellaceae sp. 1 (BF7) to 28% in Teratosphaeriaceae sp. (BF9) (Figure 2). Overall, this correlates with previous findings that describe an average percentage of <1%–30% transposable elements within fungal genomes,^124^ although partially some samples (BF1, BF3, BF9, BF10, BF11, BF14, BF15) are above the expected maximal percentage of 5% repeat content for Ascomycota.^125^ Black fungi in the Dothideomycetes had a slightly higher proportion of repetitive elements than those in the Eurotiomycetes.

**FIGURE 3:**
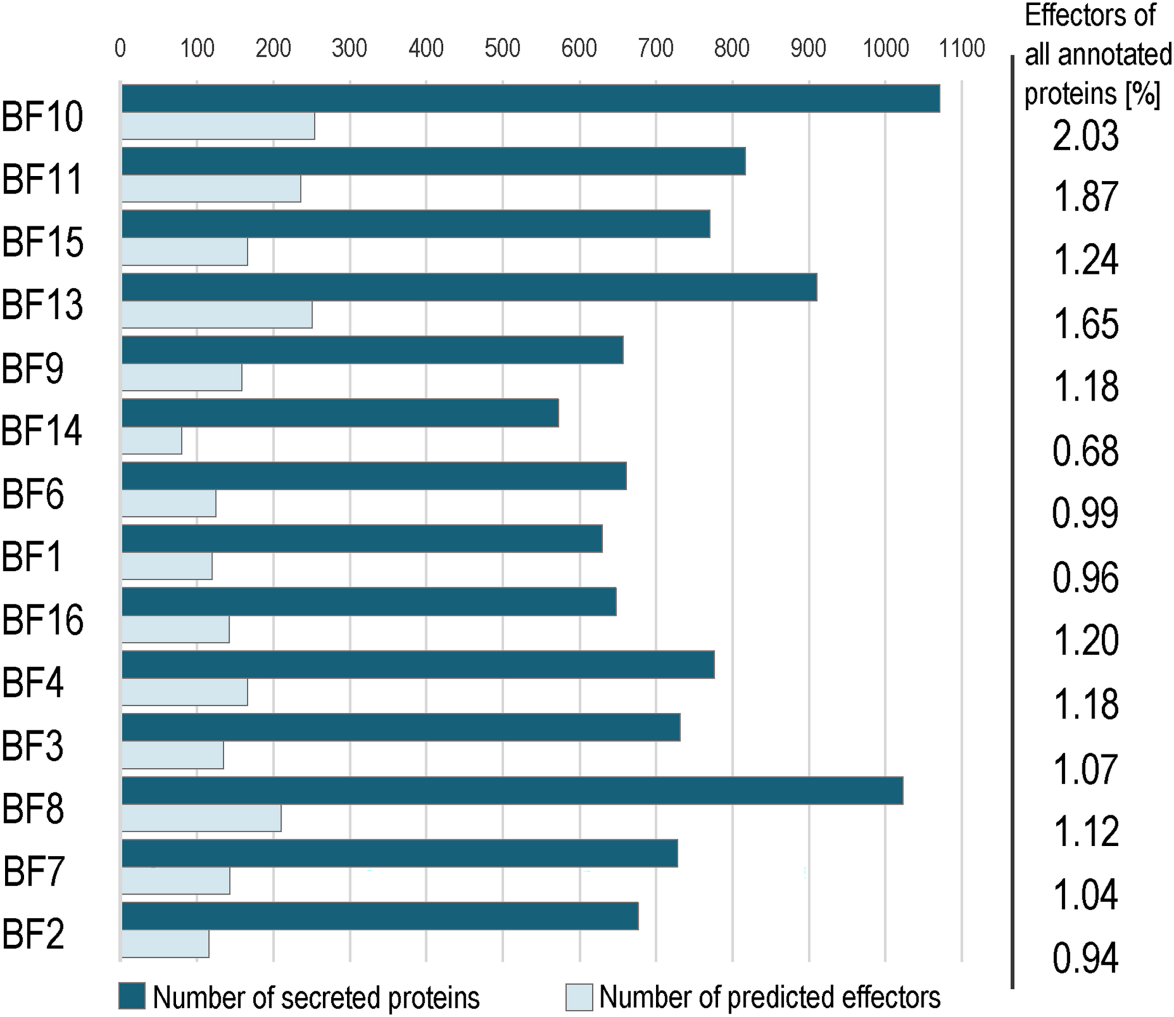
Number of predicted effectors and secreted proteins in genomes of black fungi, supplemented with the percentage of effectors in relation to all annotated proteins. Apoplastic and cytoplasmic effectors are summed up. Effectors were predicted using EffectorP.^97^

Transcriptome‐based annotations yielded ~11,000–18,000 genes and proteins per genome (Figure 2, Table S3). The functional annotation provided between ~7000–11,000 gene models with a Gene Ontology annotation (Table S3). To get insights into putative functional roles of black fungi as lichen associates, we extended the genomic analyses by identifying effector proteins. Effectors are a group of secreted proteins that will suppress defense mechanisms of, for example, host plants, to facilitate an infection.^126^ The production of effectors is usually considered as an infection strategy, frequently observed in oomycetes and phytopathogenic fungi.^126^, ^127^ Using the machine learning‐based effector prediction tool EffectorP,^97^ the number of predicted apoplastic and cytoplasmic effectors ranged between 80 and 236 of 572–1071 predicted secreted proteins per genome (Figure 3, Table S3). We found that on average ~21.5% of the secreted proteins were effectors, which corresponds to 1.23% of all predicted proteins across all genomes. Although there are few conserved effectors,^128^, ^129^, ^130^ most effectors lack any conserved domain,^131^ which makes it difficult to precisely detect effector genes using only bioinformatic tools. However, machine learning tools such as EffectorP predict 81.2%–93.8%^97^ correctly. Research on phytopathogenic fungi using the same effector prediction tool yielded, for example, ~8.9% of effectors of the whole proteome and ~14.1% of all secreted proteins for *Puccinia triticana*.^132^ Ramos‐Lizardo et al.^133^ discovered similar results with an average of ~8.2% of effectors of the proteome and ~2.1% of all predicted secreted proteins for five different phytopathogenic species of the genus *Ceratocystis*. Looking into effector proteins in the mycorrhizal fungus *Rhizophagus irregularis*, ~17.8% were detected, albeit using another bioinformatic pipeline.^134^ Compared to previous studies, the average percentage of effectors of secreted proteins is higher in lichen‐associated black fungi than in phytopathogenic fungi, whereas the percentage of effectors of all predicted proteins is lower. This observation, however, might be influenced by sequencing techniques and subsequent bioinformatic analyses of predicted and secreted proteins, and hence should be interpreted with caution. Effector prediction tools such as EffectorP^97^ point out the number of expected effector proteins, without any further information about functional roles or characteristics. Further investigation is needed to clarify whether effectors play a role in the interaction of black fungi with other members of the lichen symbiosis.

### 3.3 Biosynthetic gene clusters

We detected 351 secondary metabolite gene clusters across all 14 black fungal genomes, using antiSMASH^98^ (Figure 2, Table S3). The average number of predicted clusters was ~25 per genome. The genome of Pseudopyrenochaetaceae sp. (BF11) stood out as it contained twice as many secondary metabolite gene clusters as the average genomes analyzed here, while it was not larger in genome size. Each of the genomes contained at least one type I PKS, non‐ribosomal peptide synthetase (NRPS), terpene cluster, and ribosomally synthesized and post‐translationally modified peptide.

**FIGURE 4:**
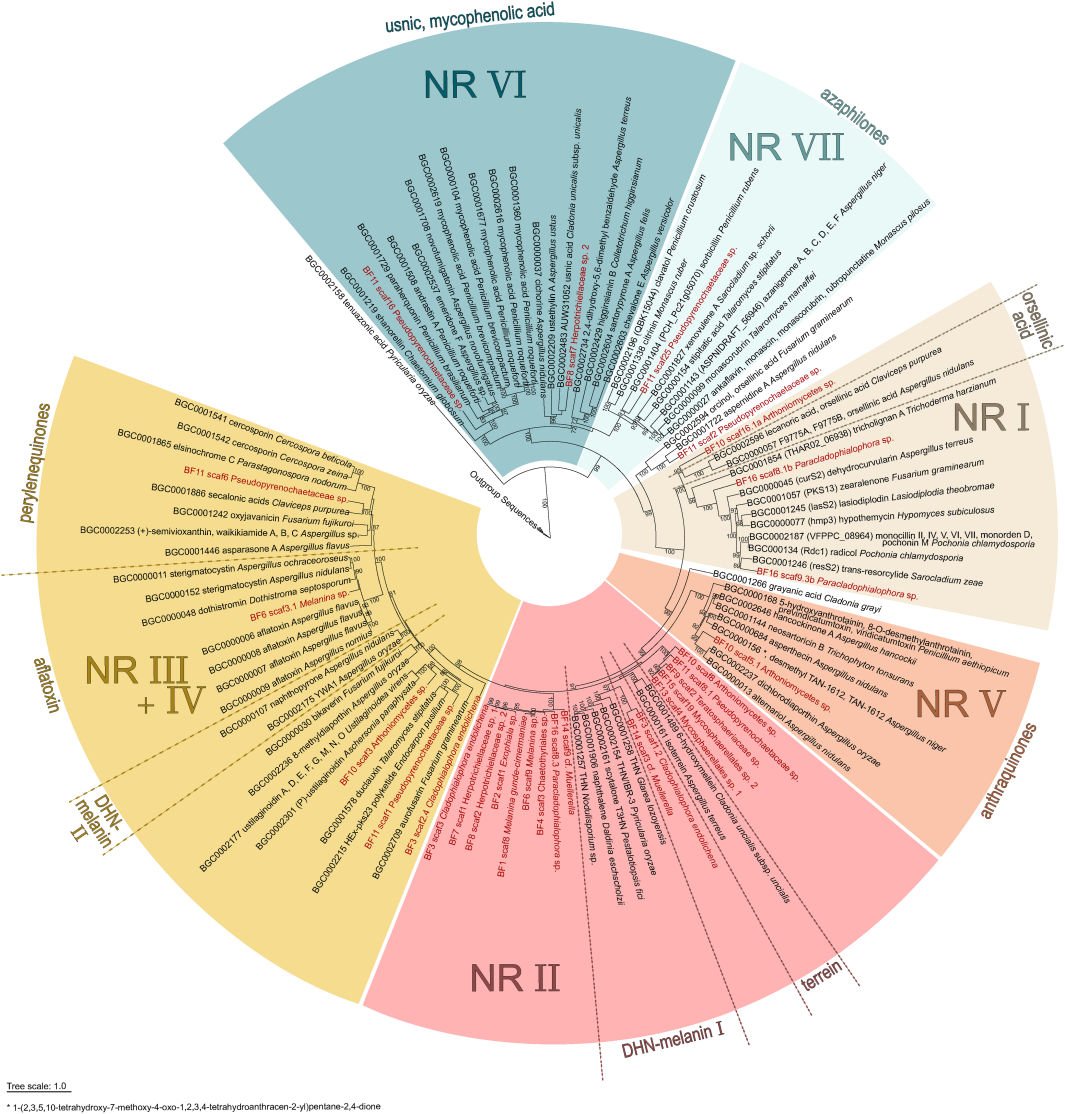
Phylogenetic relationships of the keto synthase (KS) domain of polyketide synthase genes. This is a maximum likelihood phylogeny of non‐reducing PKSs (NR‐PKSs). ML bootstrap support values ≥70% are shown, and a tree scale bar is indicated at the bottom of the tree. Black fungal isolates of the present study are highlighted in red. We included all Ascomycota NR‐PKSs linked to a secondary metabolite from the MIBiG database.^102^ Clade numbers follow previous publications.^104^, ^105^, ^106^ Example metabolites or metabolite classes are indicated.

Melanin biosynthesis is an important and unifying trait in black fungi, and here, we focused on secondary metabolite gene clusters putatively associated with melanin biosynthesis. Many fungal pigments are derived from polyketide pathways,^135^ including the common DHN‐melanin pathway.^48^ Here, we assessed whether black fungi share homologous PKS genes related to the DHN‐melanin biosynthesis. The presented phylogeny, based on the KS domain, includes overall 111 sequences: 76 NR‐PKSs of known function from the MIBiG database,^102^ 29 of black fungi that were identified within this study, and six FAS outgroup sequences (Figure 4). Secondary metabolites associated with these clades are indicated in Figure 4. NR‐PKSs from black fungi are dispersed over the phylogeny, occurring in all of the generally recognized clades.^104^, ^105^, ^106^ However, two clusters of homologous genes, both in the NR II clade, stand out. Every black fungal genome had a PKS, which belonged to either of these two groups. The NR II clade includes PKSs linked to the DHN‐melanin biosynthesis and to the synthesis of terrein and its precursor 6‐hydroxymellein.^136^ One group, including nine homologous sequences, consists only of members of the Eurotiomycetes (BF1, BF2, BF3, BF4, BF6, BF8, BF14, BF16), and the other group, comprising five sequences, consists of Dothideomycetes (BF9, BF11, BF13, BF15) and the Arthoniomycete (BF10) sequence. Our findings suggest that all 14 black fungi analyzed here possess a pathway related to DHN‐melanin biosynthesis.

## 4 CONCLUSION

Black fungi and lichenized fungi are known to live and thrive in the most inhospitable environments on Earth.^137^ Symbiotic associations as survival strategies seem therefore reasonable, as already postulated in many other studies.^14^, ^25^, ^137^, ^138^ Phylogenetic analyses of this research showed a close relationship of the herein analyzed black fungal strains to taxa that were isolated from symbiotic communities, pointing toward a phylogenetic link between lichen‐associated black fungi and other members of similar communities, hence a potential profitable relationship. Furthermore, strains of *Cladophialophora* and *Exophiala* are widely known for their ability to assimilate hydrocarbon compounds,^139^ which indirectly shows that black fungi can benefit of secondary metabolites of lichens. It is conceivable that black fungi contribute bioactive substances to the secondary metabolite cocktails found in lichen symbioses. Both lichenized and black fungi are slow‐growing organisms^140^, ^141^ and produce natural products with antimicrobial activities as defense mechanisms.^142^, ^143^, ^144^, ^145^ By growing together, both may benefit from the pool of diverse metabolites present in and on the thallus. The phylogenetically diverse taxa analyzed here share homologous PKS genes putatively linked to DHN‐melanin biosynthesis, suggesting these evolutionarily conserved genes play a crucial role in their ecology.

The new genomes of lichen‐associated black fungi constitute a valuable genomic resource, which will likely fuel research in a number of related fields, such as biogeography, host specificity, and secondary metabolism of black fungi. They provide a new resource for understanding the genomic underpinnings of environmental adaptation and mutualistic species interactions. It is a further step into unraveling the complexity of the lichen microecosystem.

## AUTHOR CONTRIBUTIONS

IS, VK, and JO conceived the ideas and designed the research. JO carried out the laboratory work. VK performed all analyses and evaluated the data with contributions from AC. VK and IS wrote the manuscript, with contributions of JO in section 2.2. All authors read and approved the final manuscript.

## CONFLICT OF INTEREST STATEMENT

The authors declare no conflict of interest.

## Supporting information


Figure S1.

Figure S2.

Figure S3.

Figure S4.

Figure S5.

Table S1.

Table S2.

Table S3.

Table S4.

Data S1.

## Data Availability

This Whole Genome Shotgun project has been deposited at NCBI GenBank under the BioProject accession ID PRJNA1083337. Genomic data have been deposited under the BioSample accession ID SAMN40245485–SAMN40245498. ITS sequences can be accessed through accessions PP463922–PP463935 and nuLSU PP463936–PP463949. All sequences used in phylogenies within this research are listed under their respective accession number in Tables S2 and S4.
