# Supplementary figures and images for: Genomic features of lichen‐associated black fungi
Source: IUBMB Life. 2024 Dec 22;77(1):e2934. doi: 10.1002/iub.2934 (PMC11664114; doi:10.1002/iub.2934)

# Eurotiomycetes

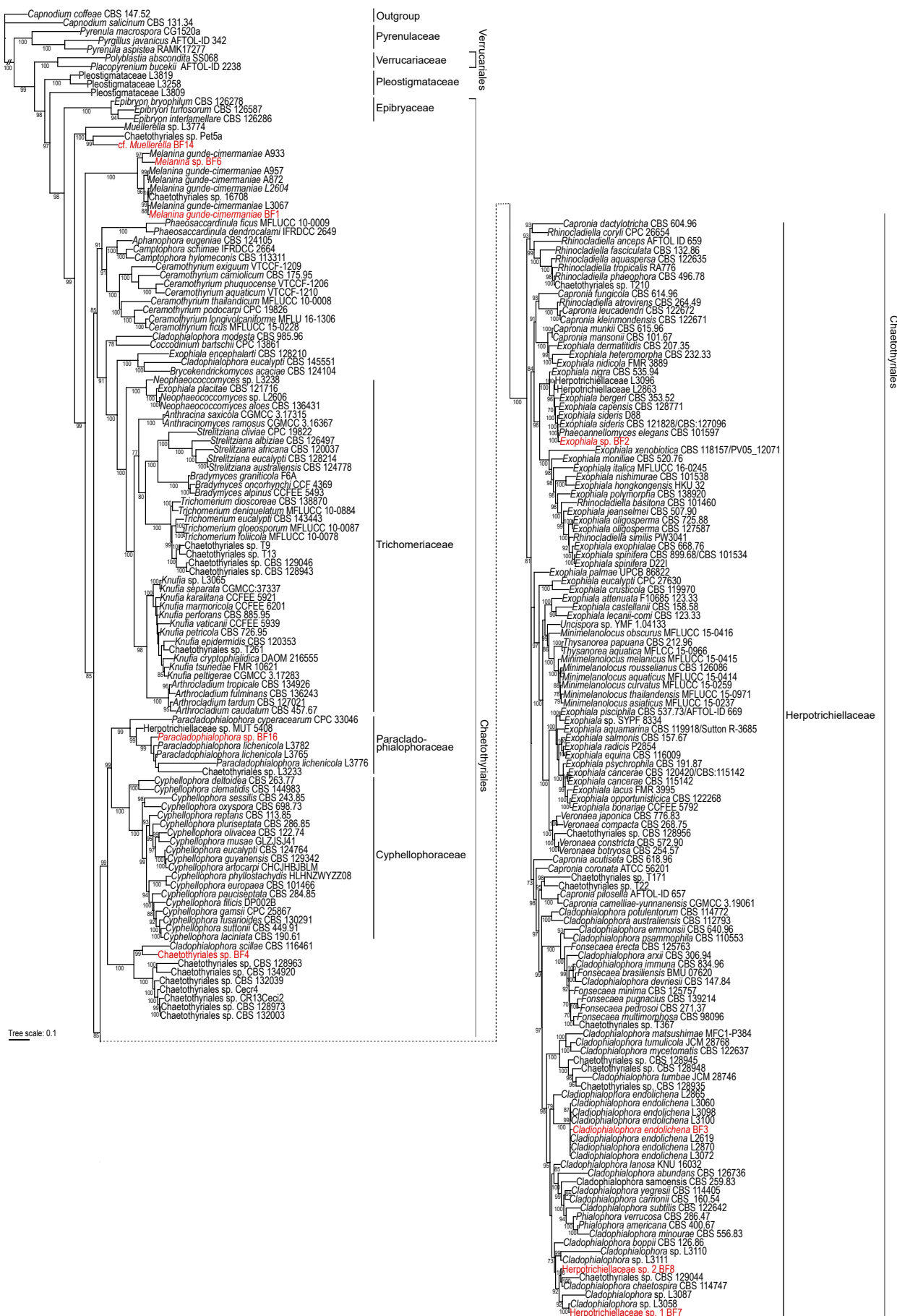

Supplement: Supplementary file 1 — Figure S1. [file IUB-77-0-s002.pdf]

# Dothideomycetes

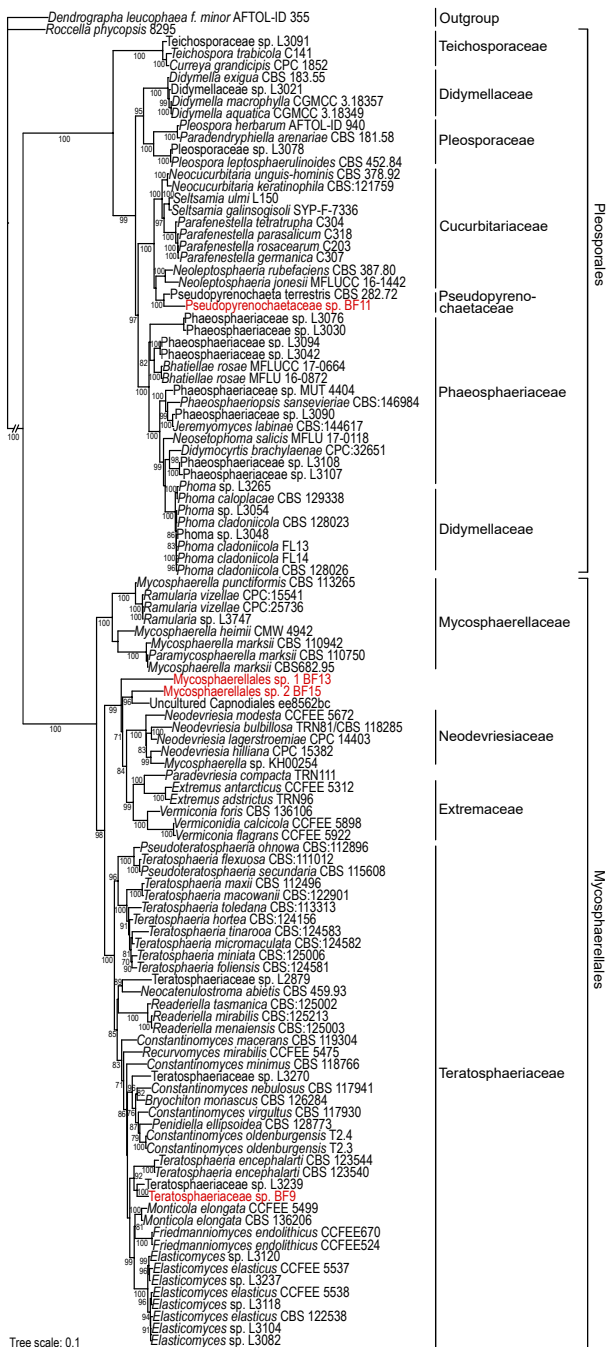

Supplement: Supplementary file 2 — Figure S2. [file IUB-77-0-s001.pdf]

# Arthoniomycetes

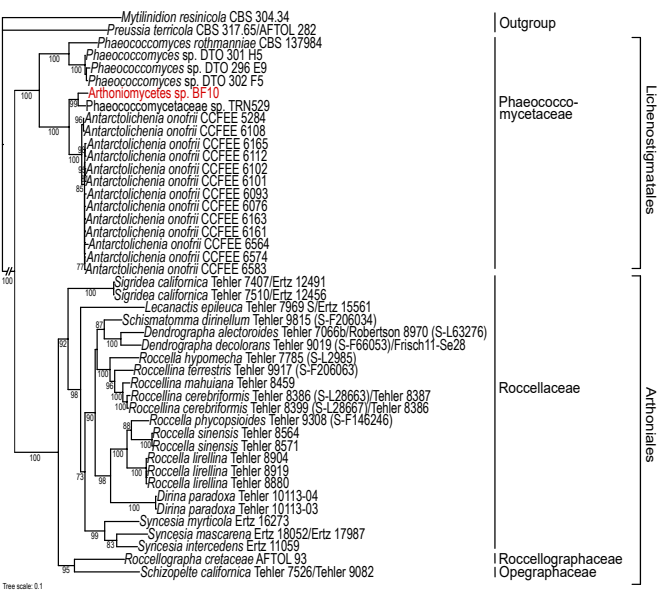

Supplement: Supplementary file 3 — Figure S3. [file IUB-77-0-s006.pdf]
