# Supplementary material for: Genomic features of lichen‐associated black fungi
Source: IUBMB Life. 2024 Dec 22;77(1):e2934. doi: 10.1002/iub.2934 (PMC11664114; doi:10.1002/iub.2934)
Supplement: Supplementary file 4 — Figure S4. [file IUB-77-0-s003.pdf]

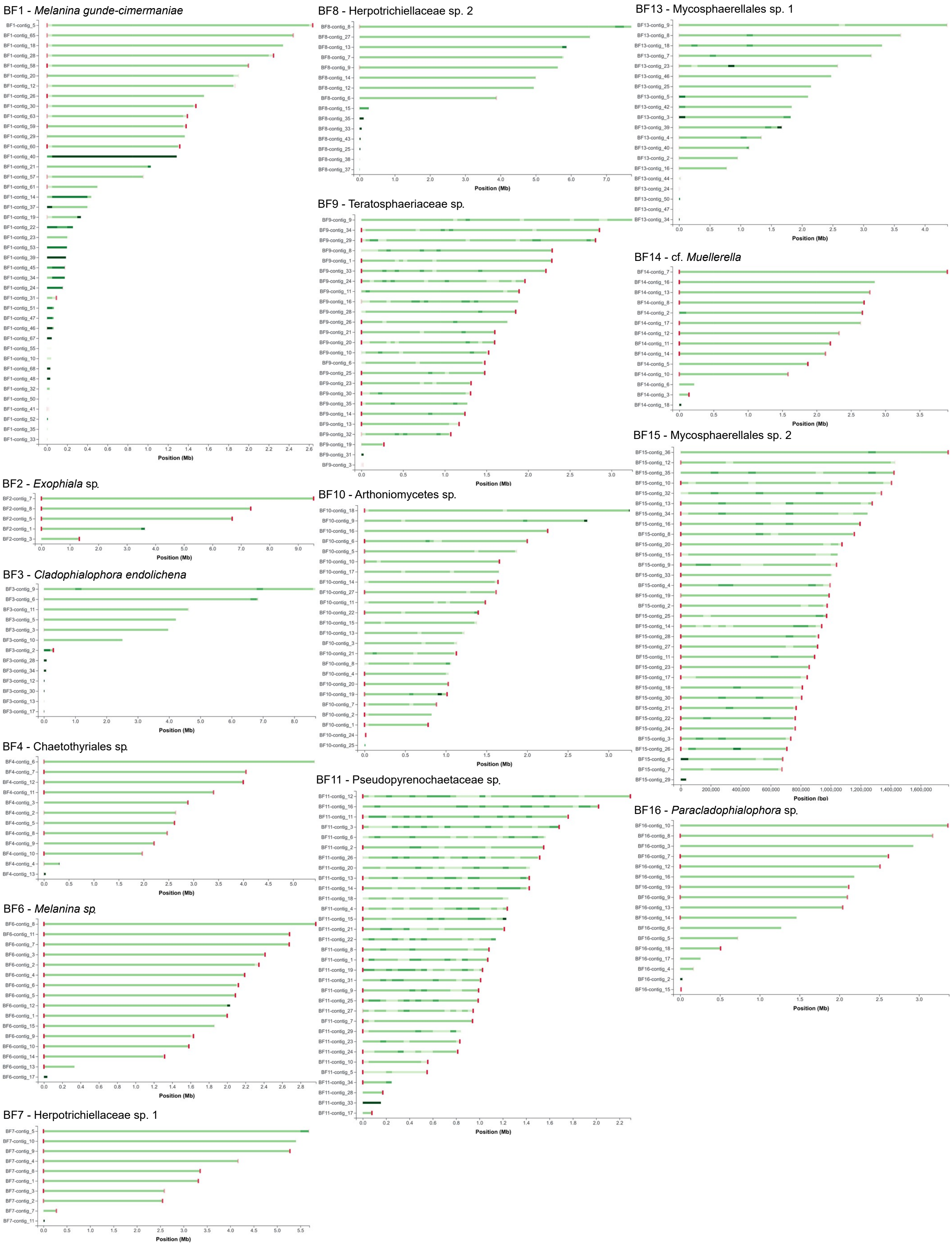
BF1 - Melanina gunde-cimermaniae
Position (Mb)
BF2 - Exophiala sp.
Position (Mb)
BF3 - Cladophialophora endolichena
Position (Mb)
BF4 - Chaetothyriales sp.
Position (Mb)
BF6 - Melanina sp.
Position (Mb)
BF7 - Herpotrichiellaceae sp. 1
Position (Mb)
BF8 - Herpotrichiellaceae sp. 2
Position (Mb)
BF9 - Teratosphaeriaceae sp.
Position (Mb)
BF10 - Arthoniomycetes sp.
Position (Mb)
BF11 - Pseudopyrenochaetaceae sp.
Position (Mb)
BF13 - Mycosphaerellales sp. 1
Position (Mb)
BF14 - cf. Muellerella
Position (Mb)
BF15 - Mycosphaerellales sp. 2
Position (bp)
BF16 - Paracladophialophora sp.
Position (Mb)
