# Supplementary material for: Genomic features of lichen‐associated black fungi
Source: IUBMB Life. 2024 Dec 22;77(1):e2934. doi: 10.1002/iub.2934 (PMC11664114; doi:10.1002/iub.2934)
Supplement: Supplementary file 5 — Figure S5. [file IUB-77-0-s005.pdf]

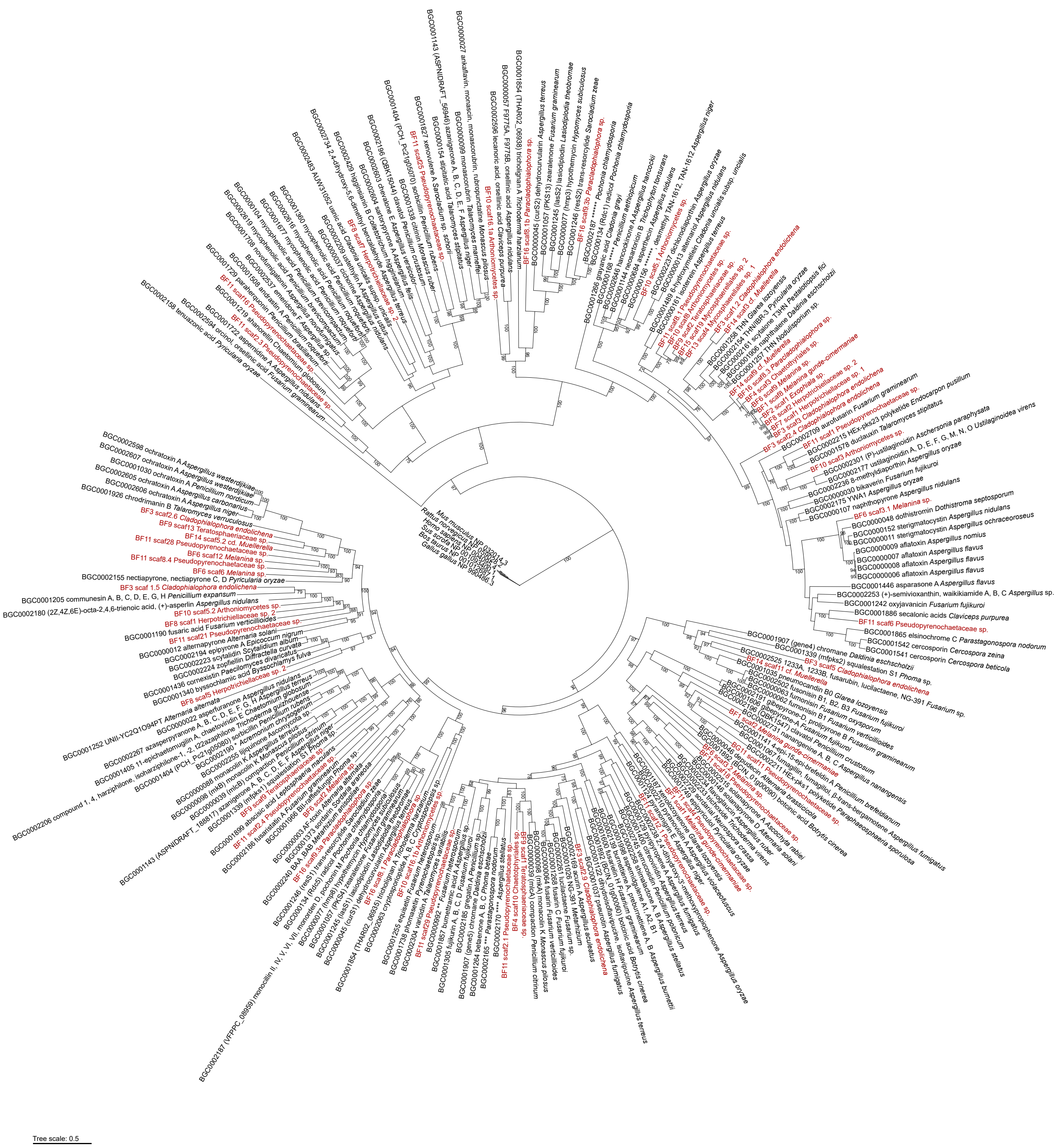

*2'3'-dihydrosorbicillin, 2'3'-dihydrosorbicillinol, bisorbicillinol, bisvertinolone (spelled bisvertinolon in paper), dihydrobisvertinolone, oxosorbicillinol, sorbicillin, sorbicillinol, tetrahydrobisvertinolone
**(3R,5S)-5-(4-hydroxybenzyl)-3-methyl-3-((2E,4E,6E,8E,10E)-4,8,10-trimethyldodeca-2,4,6,8,10-pentaenoyl)pyrrolidine-2,4-dione, fusaridione A
***betaenone C, compound 3, compound 4, compound 5, compound 6, compound 7, compound 9, dehydroprobetaenone I, probetaenone I, stemphyloxin II
****4-methyl-5-dimethyltetradecahexaene-β-ketolactone, 4-methyl-5-methylheptatriene-β-ketolactone
*****5-hydroxyanthrotainin, 8-O-desmethylanthrotainin, previridicatumtoxin, viridicatumtoxin
******(VFPPC_08964) monocillin II, IV, V, VI, VII, monorden D, pochonin M
*******1-(2,3,5,10-tetrahydroxy-7-methoxy-4-oxo-1,2,3,4-tetrahydroanthracen-2-yl)pentane-2,4-dione
