# Supplementary material for: Genomic features of lichen‐associated black fungi
Source: IUBMB Life. 2024 Dec 22;77(1):e2934. doi: 10.1002/iub.2934 (PMC11664114; doi:10.1002/iub.2934)
Supplement: Supplementary file 10 — Data S1. [file IUB-77-0-s004.docx]

Figure legends:

Supplemental figure SF1. Maximum likelihood phylogenetic analysis based on ITS and nuLSU sequences of Eurotiomycetes. ML bootstrap support values ≥70% are shown and a tree scale bar is indicated at the bottom of the tree. Black fungal strains generated in this study are highlighted in red. Sequences retrieved from GenBank (77) are provided in supplemental table ST2 under the respective accession number.

Supplemental figure SF2. Maximum likelihood phylogenetic analysis based on ITS and nuLSU sequences of Dothideomycetes. ML bootstrap support values ≥70% are shown and a tree scale bar is indicated at the bottom of the tree. Black fungal strains generated in this study are highlighted in red. Sequences retrieved from GenBank (77) are provided in supplemental table ST2 under the respective accession number.

Supplemental figure SF3. Maximum likelihood phylogenetic analysis based on ITS and nuLSU sequences of Arthoniomycetes. ML bootstrap support values ≥70% are shown and a tree scale bar is indicated at the bottom of the tree. Black fungal strains generated in this study are highlighted in red. Sequences retrieved from GenBank (77) are provided in supplemental table ST2 under the respective accession number.

Supplemental figure SF4. Assembly contiguity of all assemblies of the isolated black fungal strains. Tapestry (92) output. Telomere sequences (TTAGGG/CCCTAA) (93, 94) are marked in red. The color intensity of the red bars reflects the number of detected telomere sequences. The number and length (in Mb or bp) of contigs are represented by the green lines. The intensity of the green color indicates the sequencing depth.

Supplemental figure SF5. Phylogenetic relationships of the keto synthase (KS) domain of polyketide synthase genes. This is a maximum likelihood phylogeny of non-reducing and reducing PKSs. ML bootstrap support values ≥70% are shown and a tree scale bar is indicated at the bottom of the tree. Black fungal isolates of the present study are highlighted in red. We included all Ascomycota PKSs linked to a secondary metabolite from the MIBiG database (102). PKSs clustering into a separate clade consisting of fatty acid synthases (FAS) were removed from the alignment.

Table legends:

Supplementary table ST1. Collection data of lichen individuals.
Supplementary table ST2. NCBI sequence accession numbers.
Supplementary table ST3. Extended genome content overview.
Supplementary table ST4. MIBiG and NCBI sequence accessions for NR-PKS
phylogenies.
